# High isolation 16-port massive MIMO antenna based negative index metamaterial for 5G mm-wave applications

**DOI:** 10.1038/s41598-023-50544-z

**Published:** 2024-01-02

**Authors:** Alya Ali Musaed, Samir Salem Al-Bawri, Wazie M. Abdulkawi, Khaled Aljaloud, Zubaida Yusoff, Mohammad Tariqul Islam

**Affiliations:** 1 Space Science Centre, Climate Change Institute, Universiti Kebangsaan Malaysia (UKM), 43600 Bangi, Malaysia; 2 Department of Electrical Engineering, Collegeof Engineering in Wadi Addawasir, Prince Sattam Bin Abdulaziz University, Al-Kharj, Saudi Arabia; 3 College of Engineering, Muzahimiyah Branch, King Saud University, 11451 Riyadh, Saudi Arabia; 4 Faculty of Engineering, Multimedia University, 63100 Cyberjaya, Selangor Malaysia; 5 Department of Electrical, Electronic and Systems Engineering, Faculty of Engineering and Built Environment, Universiti Kebangsaan Malaysia UKM, 43600 Bangi, Selangor Malaysia

**Keywords:** Engineering, Materials science

## Abstract

A 16-port massive Multiple-Input-Multiple-Output (mMIMO) antenna system featuring a high gain and efficiency is proposed for millimeter-wave applications. The antenna system consists of 64 elements with a total size of 17 λ***o*** × 2.5λ***o***, concerning the lowest frequency. Each 2 × 2 (radiating patch) subarray is designed to operate within the 25.5–29 GHz frequency range. The antenna's performance in terms of isolation, gain, and efficiency has been significantly improved by utilizing the proposed unique double and epsilon negative (DNG/ENG) metamaterials. The array elements are positioned on top of a Rogers RT5880 substrate, with ENG metamaterial unit cells interposed in between to mitigate coupling effects. Additionally, the DNG metamaterial reflector is positioned at the rear of the antenna to boost the gain. As a result, the metamaterial-based mMIMO antenna offers lower measured isolation reaching 25 dB, a maximum gain of 20 dBi and an efficiency of up to 99%. To further analyze the performance of the MIMO antenna, the diversity gain and enveloped correlation coefficient are discussed in relation to the MIMO parameters.

## Introduction

Nowadays, mm-wave massive multiple-input-multiple-output (massive MIMO) system is considered as one of the most prominent enablers for 5G and beyond wireless communication systems^1,2^. However, interelement mutual coupling is a significant obstacle in antenna array system construction. When a large number of antennas are placed in close proximity to one another, performance degradation due to insufficient isolation is the primary concern. Decoupling network^3,4^, defected ground structure^5–9^ self-isolated antenna^10^ and metamaterials^9,11–14^ are a few of the remarkable techniques proposed by researchers to enhance isolation and produce a low-profile, highly effective antenna system. Nevertheless, due to their distinctive properties, metamaterials have attracted the interest of a large number of scientists. Multiple studies^13,15–17^ have described mm-wave MIMO antennas employing metamaterial structures to enhance antenna performance, particularly mutual coupling reduction or antenna gain. Few literary works have been written about mMIMO antenna designs. As Is^18–20^, the majority of these solutions are based on an array mode with gain and isolation capabilities. 64 RF elements were created using standard, 3.5 GHz-designed 4 × 1 subarrays of patches in^18^. The design was built and tested, and the results showed that the entire array had a gain of 18 dBi at boresight and a bandwidth (BW) of 200 MHz. 108 antenna patches on a single planar size made up the array presented in^19^ with a 183 MHz BW, the system consists of 12 patches operating at a frequency of 3.5 GHz and 96 patches operating at a frequency of 26 GHz. The array antenna shown in^20^ had a maximum coupling of 35 dB between its ports and covered a range of 250 MHz whereas gain was measured to be 10.6 dB.

In this article, we suggest a wideband massive MIMO antenna system that operates within the mm-wave frequency band. DNG/ENG metamaterials are employed as unit cells positioned between the antenna elements, as well as in an array configuration at the back of the antenna system, to enhance its overall performance. This metamaterial-based massive MIMO antenna system offers a lower measured isolation of 25 dB, a maximum gain of 20 dBi, and an efficiency of up to 99%. The measured and simulated findings demonstrate favorable agreement. The proposed antenna system has the potential to improve capacity and performance in 5G communication systems. To the best of author’s knowledge, this is the first instance of a massive MIMO antenna system utilizing DNG/ENG metamaterials effectively to enhance the three primary antenna parameters of isolation, gain, and efficiency.

## Configuration of metamaterial

**Figure 1:**
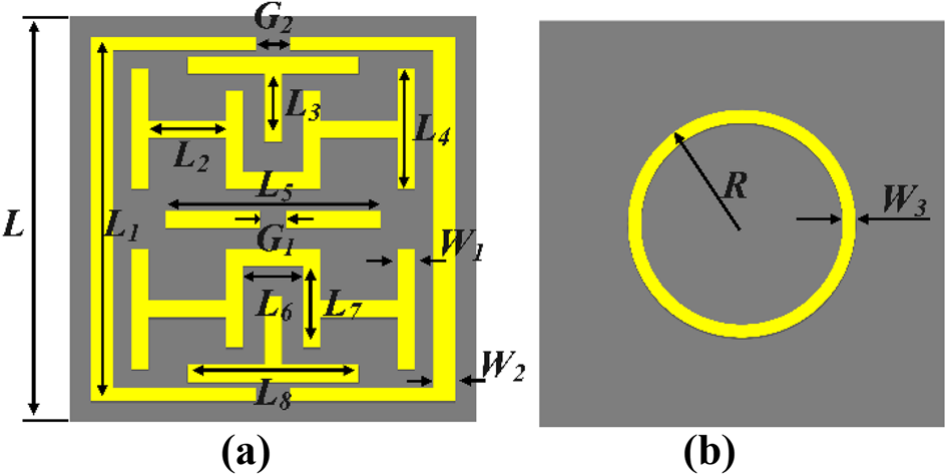
Geometry configuration of metamaterial: (**a**) front view (**b**) bottom view.

**Figure 2:**
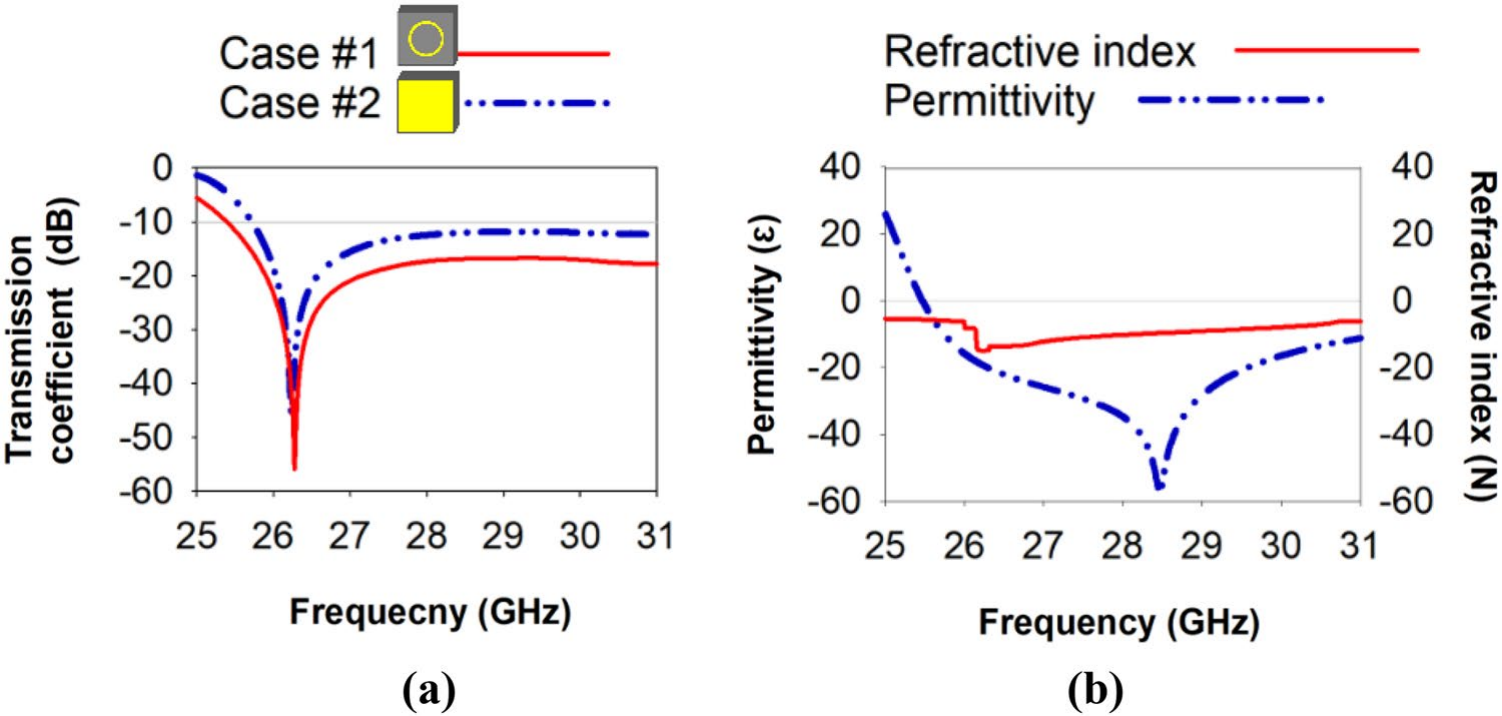
Metamaterial: (**a**) Transmission coefficient (**b**) Characteristic.

Figure 1 depicts the configuration of the metamaterial unit cell. MTM consists of a symmetric resonator in the shape of an H. The symmetric resonating patch is partitioned into four equal quartiles, each containing an H-shaped resonator, Table 1 demonstrates the MTM parameters values. Two cases of metamaterials operating at the same frequencies and exhibiting different characteristics are utilized. In the first case, the metamaterial has a circular resonator positioned at the back, demonstrating DNG characteristics within the frequency range of 25.8 to 31 GHz. In the second case, the metamaterial is completely grounded and possesses the property of negative permittivity (ENG) in the frequency band under investigation, as seen Fig. 2.

**Table 1:** Metamaterial parameters.

Parameter	Value (mm)	Parameter	Value (mm)	Parameter	Value (mm)
L	4.5	L5	2.5	G2	0.4
L1	4.25	L6	0.7	W1	0.2
L2	0.9	L7	1.05	W2	0.25
L3	0.8	L8	2	W3	0.2
L4	1.5	G1	0.3	R	1.8

The Nicolson-Ross-Weir (NRW) approach^21^, is employed to derive the effective parameters (permittivity, permeability, and refractive index) of the unit cell based on simulated data.

1 $${{\text{S}}}_{11}= \frac{{{\text{R}}}_{1}(1-{{\text{e}}}^{-2\mathrm{j\theta }})}{(1-{{\text{R}}}_{1}^{2}{{\text{e}}}^{-2\mathrm{j\theta }})}$$

2 $${{\text{S}}}_{12}= \frac{{{\text{e}}}^{-2\mathrm{j\theta }}(1-{{\text{R}}}_{1}^{2})}{(1-{{\text{R}}}_{1}^{2}{{\text{e}}}^{-2\mathrm{j\theta }})}$$

3 $${\upvarepsilon }_{{\text{r}}}\approx \frac{{\text{c}}}{\mathrm{j\pi fd}}\times \left\{\frac{1-{{\text{S}}}_{21}-{{\text{S}}}_{11}}{1+{{\text{S}}}_{21}+{{\text{S}}}_{11}}\right\}$$

4 $${\upmu }_{{\text{r}}}\approx \frac{{\text{c}}}{\mathrm{j\pi fd}}\times \left\{\frac{1-{{\text{S}}}_{21}+{{\text{S}}}_{11}}{1+{{\text{S}}}_{21}-{{\text{S}}}_{11}}\right\}$$

$${{\text{n}}}_{{\text{r}}}\approx \sqrt{{\upvarepsilon }_{{\text{r}}}{\upmu }_{{\text{r}}}}$$

5 $${{\text{n}}}_{{\text{r}}}\approx \frac{{\text{c}}}{\mathrm{j\pi fd}}\times \sqrt{\left\{\frac{{({{\text{S}}}_{21}-1)}^{2}-{{\text{S}}}_{11}^{2}}{{({{\text{S}}}_{21}+1)}^{2}-{{\text{S}}}_{11}^{2}}\right\}}$$

In the given context, the symbols used represent the following quantities: 'f' denotes frequency, 'j' represents the imaginary operator, 'd' signifies the thickness of the dielectric substrate material, 'R1' denotes the normal impedance, 'c' represents the velocity of light, and 'θ' signifies the polar angle.

## MIMO antenna design and specification

The single port microstrip patch array is made up of separate components that receive signals through microstrip feed lines attached to the patches' borders. The array antenna is made up of four rectangular antenna components placed on top of a Rogers RT5880 substrate with a full ground plane underneath. The feed lines are printed on the same side of the substrate as the patch components, and the microstrip array is powered by a corporate network. This antenna's four patches are supplied from a single point. Mitered sections are used to compensate for mismatch produced by reflections from discontinuities at 90-degree corners and T-junctions. In addition, quarter wave transformers are used to provide some impedance matching.

**Figure 3:**
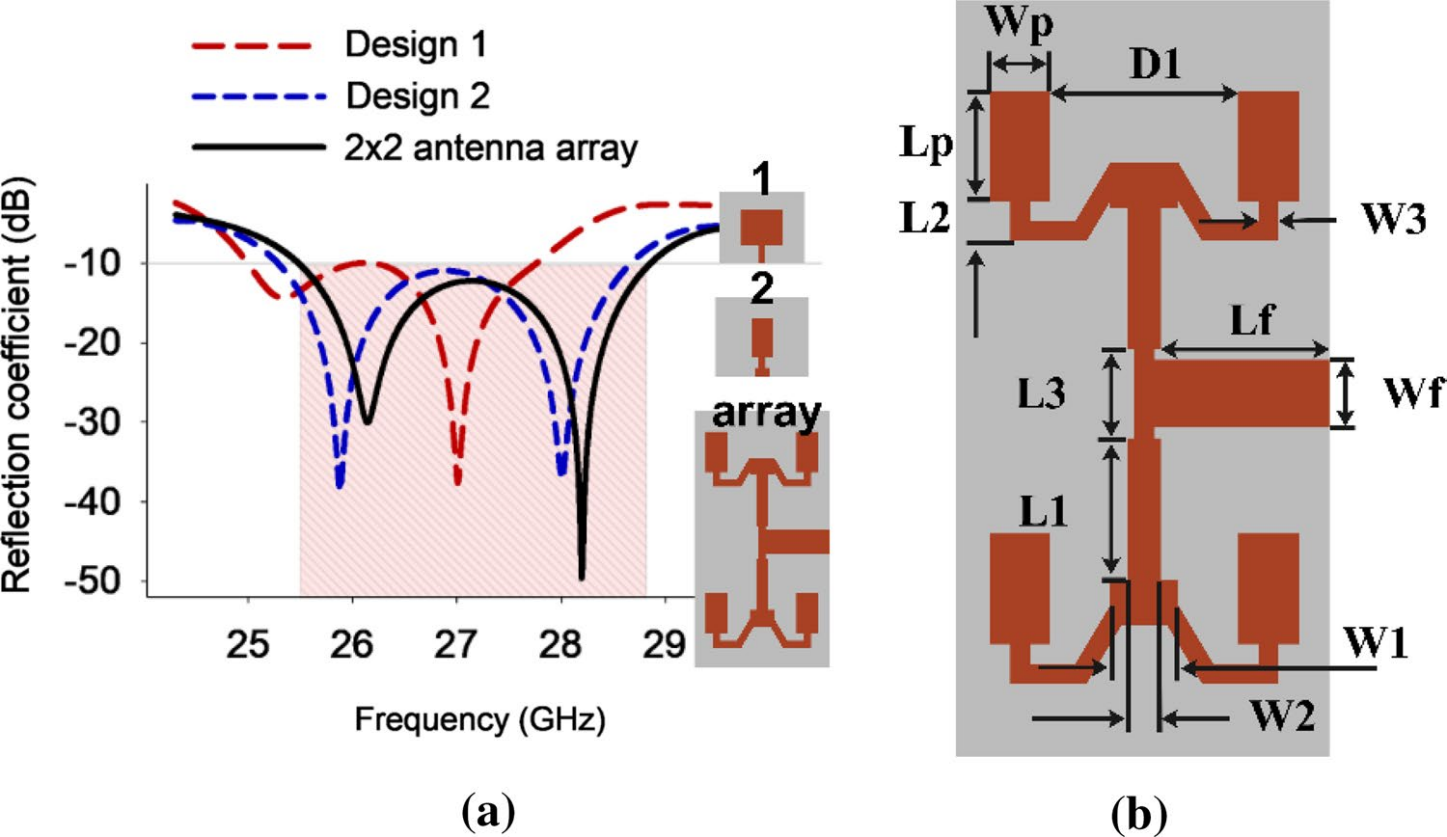
(**a**) Reflection coefficient of antenna system evolution process (**b**) Single port antenna array's dimension.

As Fig. 3a presents, the microstrip patch antenna in Design 1 that has dual resonances at 25.4 and 27 GHz and a limited bandwidth of 0.5 and 1.04 GHz, respectively. The rectangular patch in Design 2 expands the spectrum to 3.4 GHz while varying the resonance to 26 and 28 GHz. A 2 × 2 array is created, which produces equivalent results with slightly altered resonance. Figure 3b depicts the antenna structure, while Table 2 provides a comprehensive list of the antenna dimensions.

**Table 2:** Detailed dimensions of the antenna structure.

Parameter	Wp	Lp	Wf	Lf	W1	W2	W3	L1	L2	L3	D1	W	L	gab
Value (mm)	2.2	4	2.46	6.34	2.46	1.2	0.72	5.1	1.43	3.2	6.8	30	200	8.5

### Proposed 16-port massive MIMO array design

**Figure 4:**
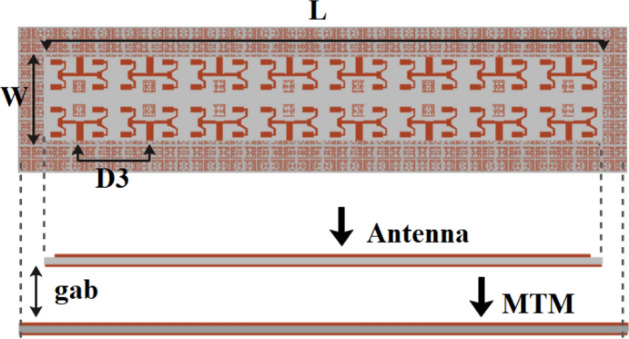
Proposed 16-port mMIMO antenna array system.

**Figure 5:**
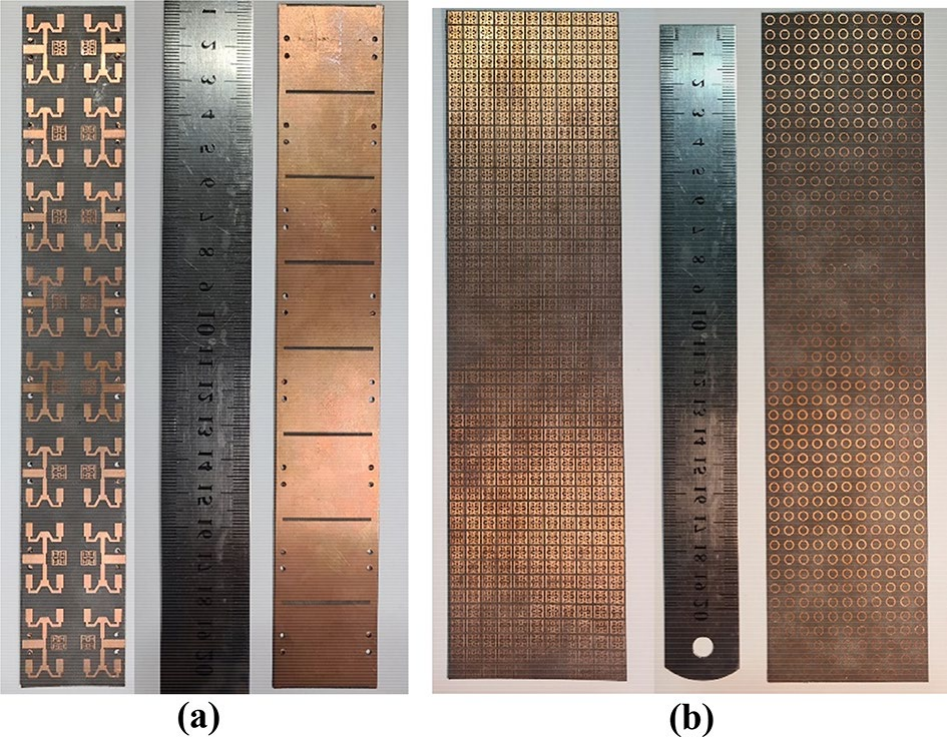
Top and bottom view of fabricated: (**a**) 16-port MIMO antenna system (**b**) metamaterial.

A 16-port mMIMO antenna system is then built to serve the future 5th-generation application. A 2 × 8 mMIMO planner antenna, with each subarray comprising of 4 elements, is proposed. All 16-port mMIMO systems share a common ground plane, which is crucial in implementing practical mMIMO antenna systems. The antennas that are positioned horizontally (across antennas) have a distance of 30 mm, denoted by (W), between their respective ports, whereas the antennas positioned vertically have a port-to-port distance of 22.5 mm, denoted by (D3), as seen in Fig. 4 A slot measuring 1 × 26 mm^2^ in dimension is situated at the ground of these antennas to augment their isolation capabilities. Moreover, a DNG metamaterial reflector was constructed using a periodic arrangement of 506 metal cells in an 11 × 46 configuration on a thin substrate of Rogers RT5880. The DNG reflector was positioned at a distance of 8 mm behind the antenna system and demonstrated high efficiency in reflecting backward waves, which allowed for significant interaction between reflected and forward waves and resulted in a substantial gain within the operating band. Fabricated prototypes are shown in Fig. 5.

## Results and discussion

**Figure 6:**
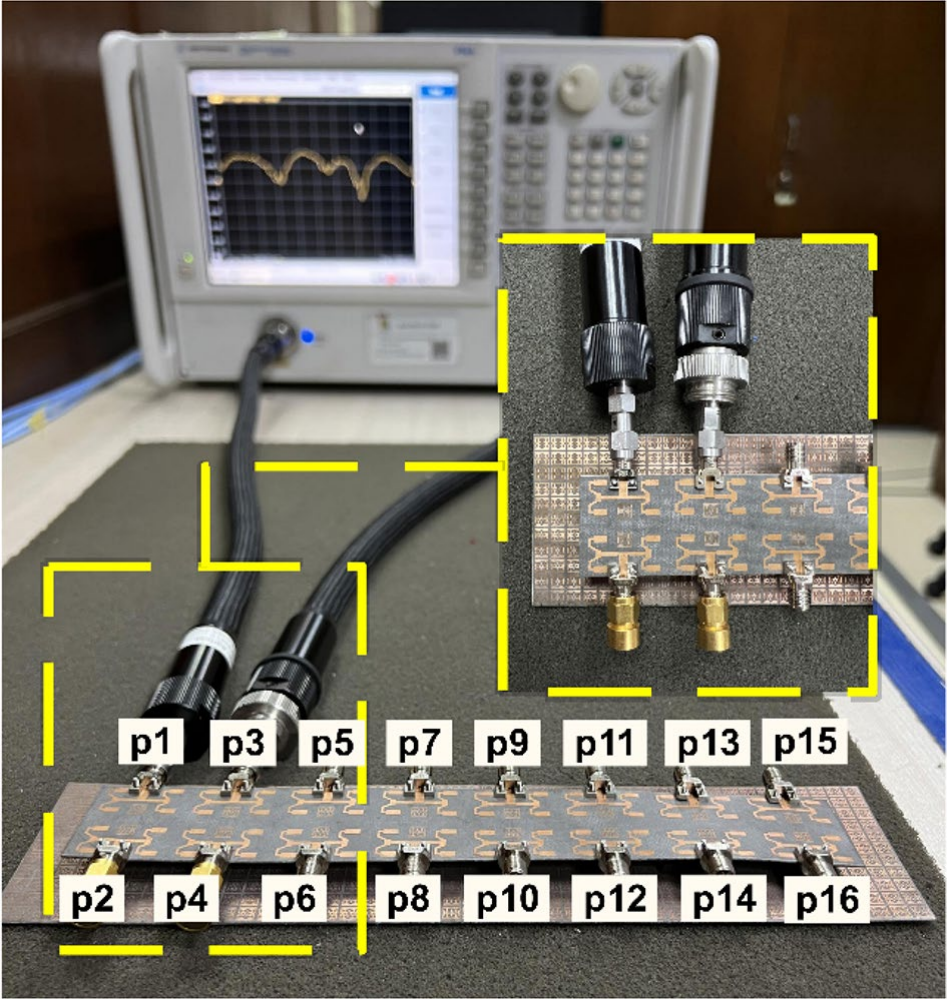
Prototype measurement setup using VNA.

The designed array was simulated using CST Studio Suite and subsequently manufactured to assess its performance. Port measurements were taken using an Agilent Vector Network Analyzer (VNA) and radiation patterns were measured in a chamber to evaluate the effectiveness of the array. Figure 6 depicts the setup for measuring the proposed design using a VNA.

### S-parameter

**Figure 7:**
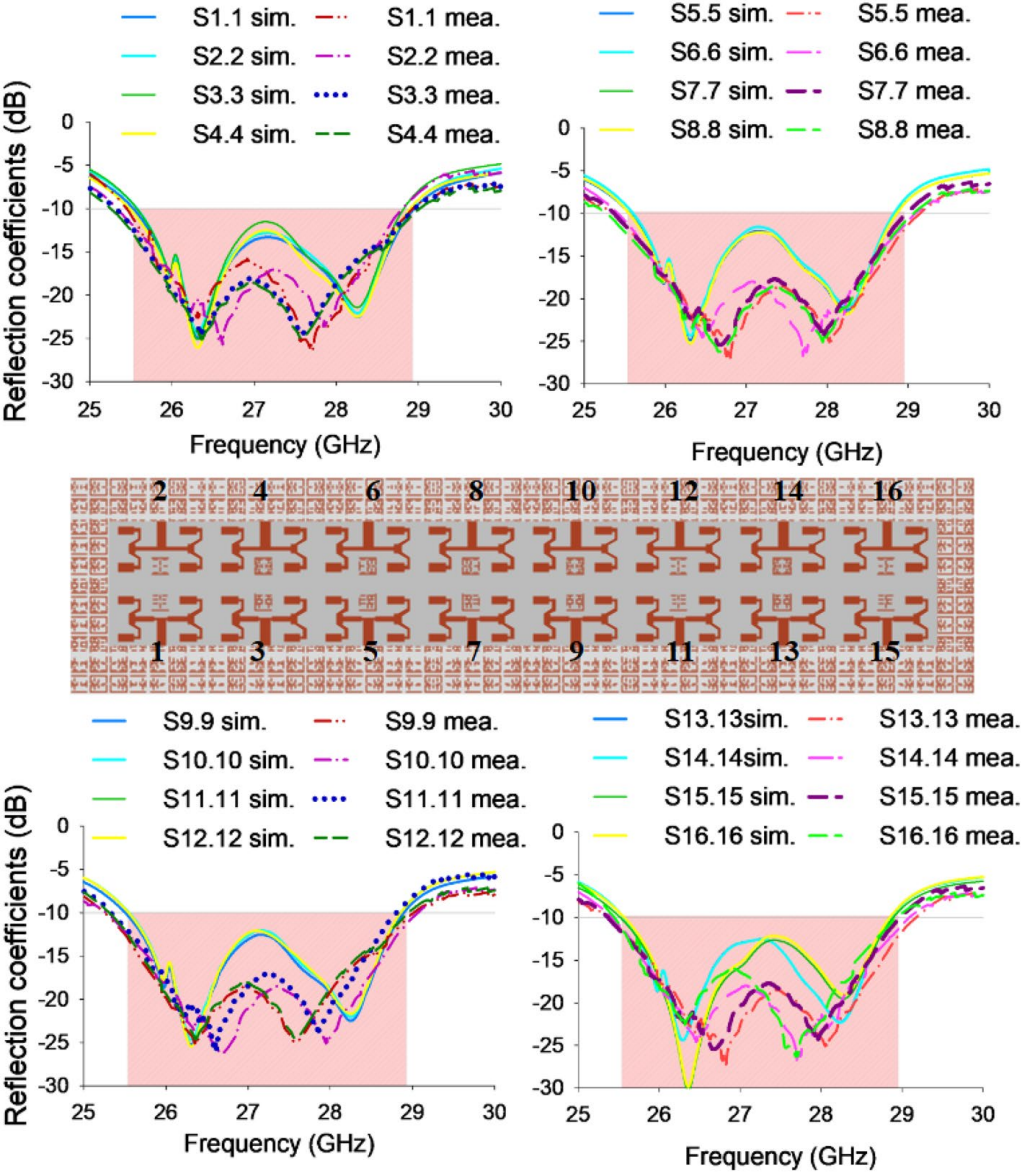
Reflection coefficient of the proposed 16-port MIMO system.

Figure 7 presents the reflection coefficient values of the 16-port system, showcasing both simulated and measured data. The findings suggest that, despite variations in their resonant frequencies, all ports demonstrate comparable characteristics within the frequency range of 25.5–29 GHz, whereas all ports effectively cover the entire subjected band.

**Figure 8:**
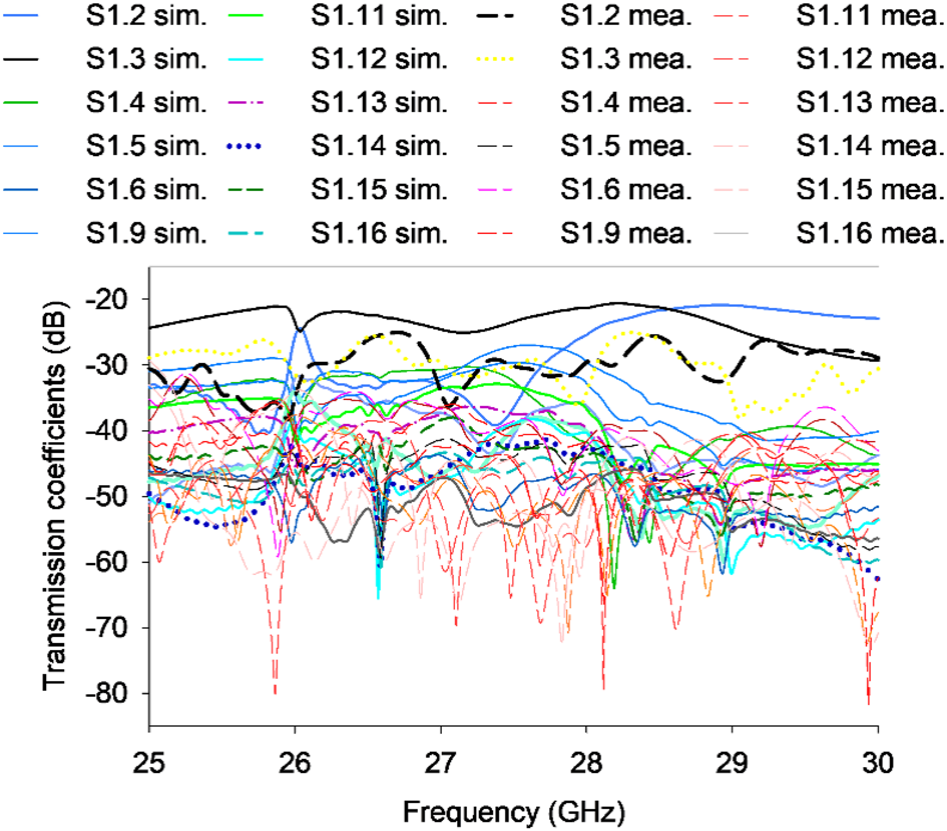
Transmission coefficient of the proposed 16-port MIMO system.

In order to emphasize the coupling effect, the proposed configuration considers only one port, as the antenna elements exhibit identical and symmetrical characteristics. The isolation values between port 1 and its adjacent ports, obtained from both simulation and measurement, are depicted in Fig. 8. The antenna system being considered demonstrates a measured isolation level exceeding 25 dB throughout the frequency spectrum ranging from 25.5 to 29 GHz, while the simulation predicts a lower isolation level, with a difference of 5 dB.

### Surface current distribution

**Figure 9:**
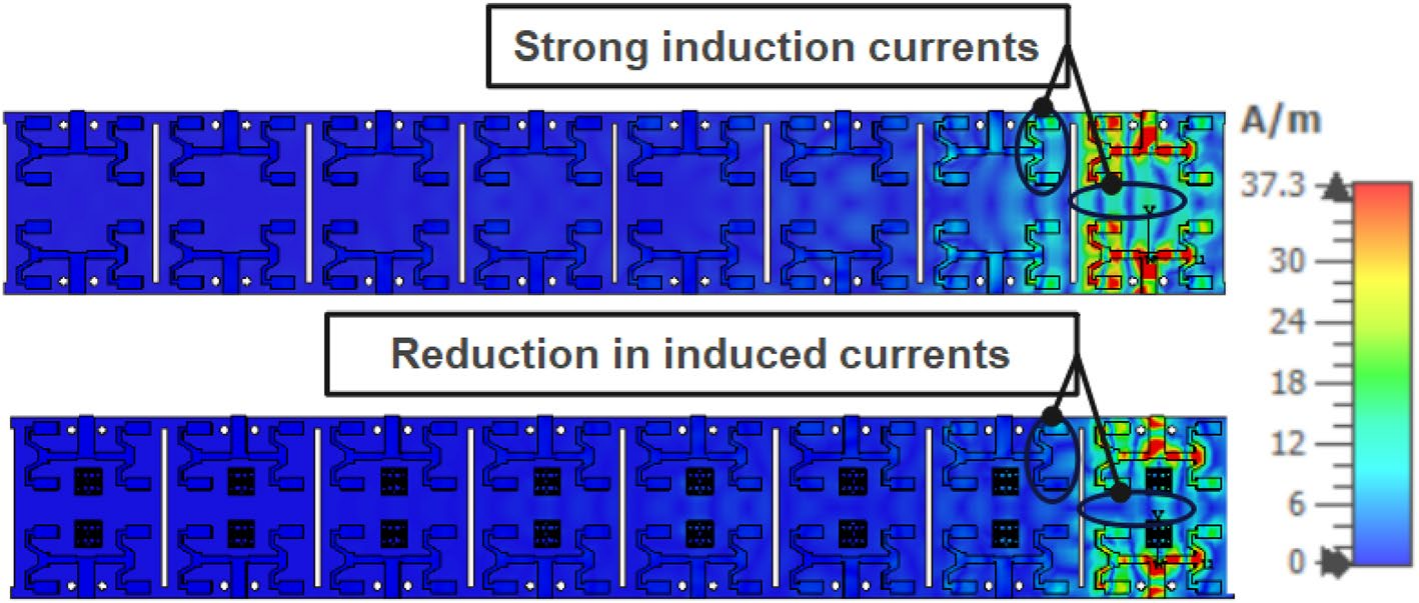
Current distribution of 2 × 2 MIMO system at 28 GHz with and without metamaterial.

Figure 9 depicts the distribution of current on the surface of the MIMO antenna system. MIMO elements can incur significant induction currents owing to electromagnetic fields created by neighboring antennas in the absence of an MTM decoupling array. These induction currents can cause undesired radiation and interference, reducing system performance. However, implementing a metamaterial structure mitigates the intense induced currents on the adjacent MIMO elements.

### Gain and efficiency

**Figure 10:**
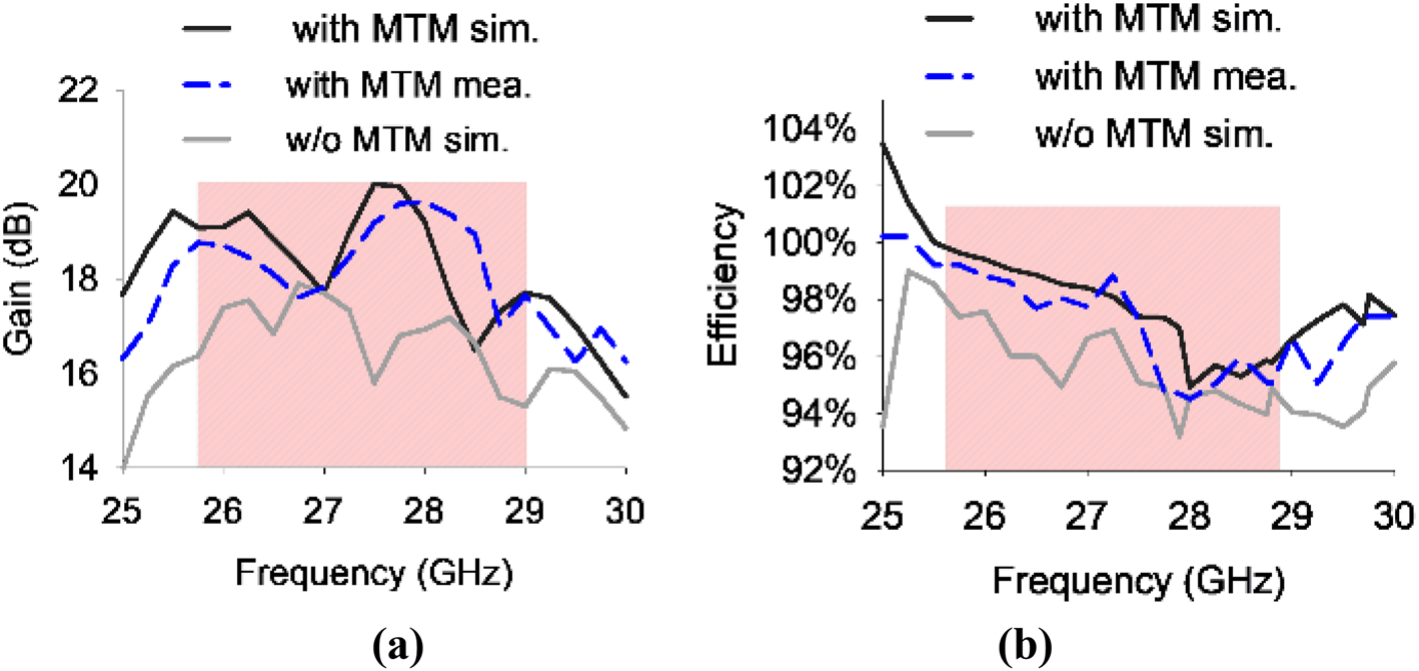
(**a**) Gain (**b**) Radiation efficiency of the proposed antenna system with and without metamaterial.

Figure 10 depicts the gain and radiation efficiency of the antenna system, both with and without the incorporation of the metamaterial. The results suggest that the integration of the metamaterial into the antenna system results in an increase in both gain and radiation efficiency. The maximum gain value observed was 19.9 dBi at 27.7 GHz with 98% radiation efficiency. The aforementioned progress highlights the ability of metamaterial in enhancing the performance of the antenna system.

Table 3 presents the performance evaluation of the proposed MIMO antenna system, comparing its performance with and without the incorporation of MTMs. The implementation of MTM techniques resulted in a significant enhancement in isolation, with an increase of 5 dB. Additionally, there was an improvement in gain, with a rise of 1.9 dB, and an increase in efficiency by 1.5%.

**Table 3:** Performance comparison.

	Isolation (dB)	Gain (dB)	Efficiency (%)
Without MTM	20	18	97.5
With MTM	25	19.9	99

### Diversity performance of MIMO antenna

The evaluation of the diversity performance of the MIMO system involves the utilization of two metrics, namely the Envelope Correlation Coefficient (ECC) and Diversity Gain (DG). In an ideal scenario, when the MIMO antenna system exhibits no correlation, the ECC should ideally have a value of zero. However, it is widely accepted in practical MIMO communication systems that ECC values equal to or less than 0.5 are considered to be acceptable for ensuring satisfactory operation^22^. Equation (6) provides a mathematical representation for calculating ECC in MIMO configurations using radiation pattern information^23^.

6 $${{\text{ECC}}}_{{\text{qp}}}=\frac{{\left|{\int }_{0}^{2\pi } {\int }_{0}^{\pi }\left({{\text{E}}}_{\theta {\text{p}}}^{*}{{\text{E}}}_{\theta {\text{q}}}{\text{XPR}}+{{\text{E}}}_{\theta {\text{p}}}^{*}{{\text{E}}}_{\theta {\text{q}}}{{\text{P}}}_{\varphi }\right)\mathrm{d\Omega }\right|}^{2}}{\alpha \times \beta }$$

$$where \;\alpha ={\int }_{0}^{2\pi } {\int }_{0}^{\pi }\left({{\text{E}}}_{\theta {\text{q}}}^{*}{{\text{E}}}_{\theta {\text{q}}}{\text{XPR}}+{{\text{E}}}_{\theta {\text{q}}}^{*}{{\text{E}}}_{\theta {\text{q}}}{{\text{P}}}_{\varphi }\right)\mathrm{d\Omega }$$ and $$\beta ={\int }_{0}^{2\pi } {\int }_{0}^{\pi }\left({{\text{E}}}_{\theta {\text{p}}}^{*}{{\text{E}}}_{\theta {\text{p}}}{\text{XPR}}+{{\text{E}}}_{\theta {\text{p}}}^{*}{{\text{E}}}_{\theta {\text{p}}}{{\text{P}}}_{\varphi }\right)d\Omega$$

**Figure 11:**
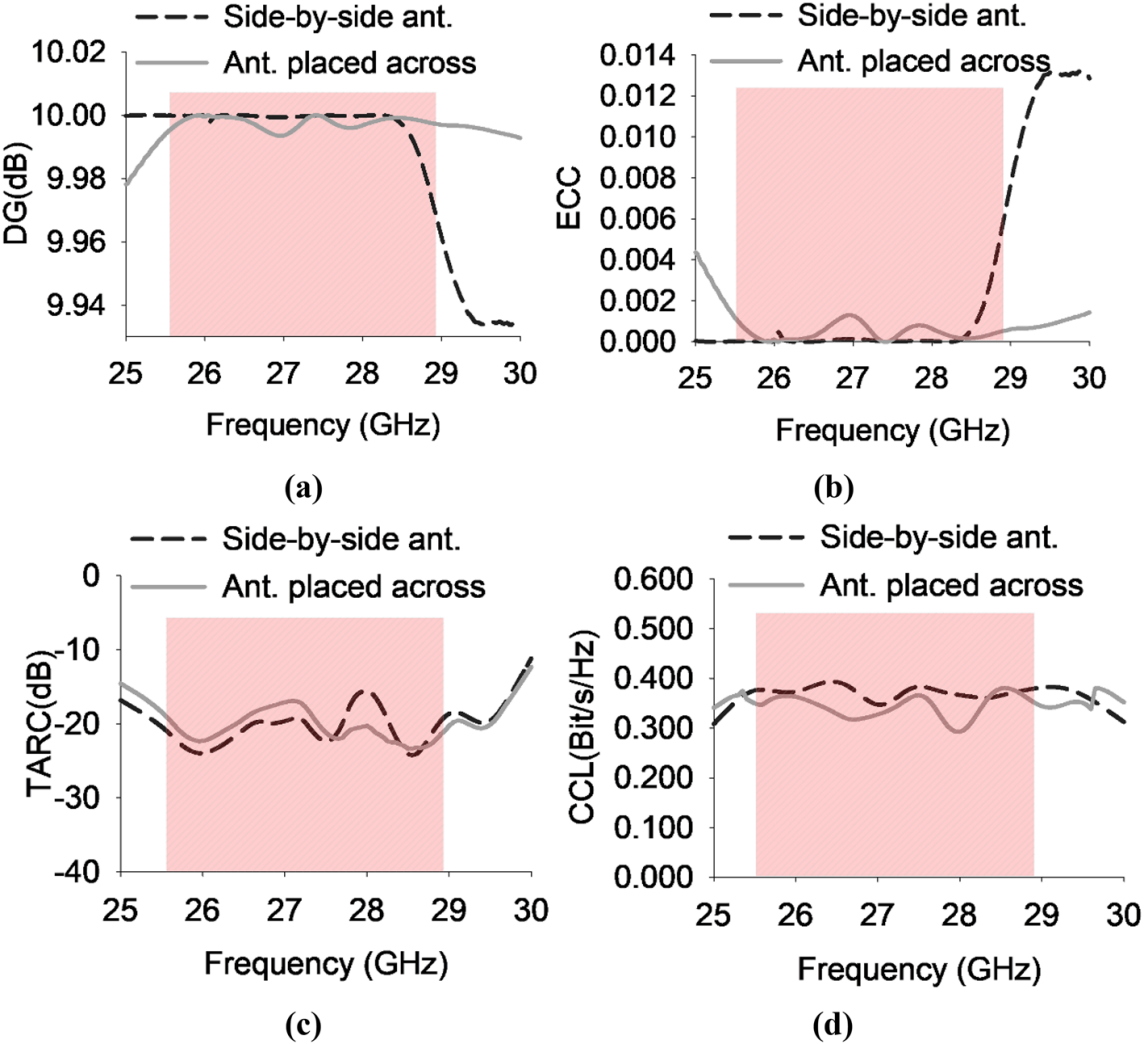
(**a**) Diversity gain (**b**) Enveloped correlation coefficient (**c**) Total active reflection coefficient and (**d**) Channel capacity loss of the proposed 16-port MIMO antenna system.

The MIMO antenna being considered exhibits an ECC value below 0.008 in its designated operating frequency range, which is notably lower than the threshold for achieving exceptional diversity performance, as depicted in Fig. 11a.

The diversity gain is an essential metric employed for assessing the efficacy of a MIMO system, and its computation can be accomplished by utilizing Eq. (7).

7 $$DG=10\sqrt{1-{ECC}^{2}}$$

Typically, a value of approximately 10 dB is considered desirable for diversity gain. Based on the ECC value of less than 0.008 for the proposed MIMO system, it can be deduced that the DG exceeds 9.99 dB within the specified operating frequency range, as depicted in Fig. 11b.

The Total Active Reflection Coefficient (TARC) is a quantitative measure employed to evaluate the ratio of power reflected from the radiating elements to the power incident on the patch in MIMO system. In order to achieve optimal efficiency in a MIMO antenna system, it is recommended to maintain a TARC value below 0 dB^24,25^. The simulated outcomes of TARC are depicted in Fig. 11(c), illustrating satisfactory performance across the whole frequency spectrum. The TARC values exhibit a consistent decrease below -14 dB for both the side by side configuration and the antenna positioned across. The TARC can be mathematically represented by the following equation:

8 $$TARC=\frac{\sqrt{\left({\left|\left({S}_{11}+\right){S}_{12}^{ej\theta }\right|}^{2}+{\left|\left({S}_{21}+\right){S}_{22}^{ej\theta }\right|}^{2}\right)}}{\sqrt{2}}$$

The primary objective of Channel Capacity Loss (CCL) is to identify the optimal transmission capacity of a communication link while minimizing data loss. In order to achieve an effective MIMO antenna system, it is preferable for the CCL to be less than 0.5 bits per second per Hertz. Equation (9) provides a mathematical representation of the CCL, incorporating the utilization of S-parameters as described in reference^7^. The MIMO antenna, as depicted in Fig. 11d, demonstrates CCL values that are lower than 0.4 bits/s/Hz.

$$CCL=-{\mathit{log}}_{2}det({\vartheta }^{\mu })$$

where

$${\vartheta }^{\mu }=\left[\begin{array}{cc}{\xi }_{11}& {\xi }_{12}\\ {\xi }_{21}& {\xi }_{22}\end{array}\right]$$

$${\xi }_{11}=1-\left[{\left|{S}_{11}\right|}^{2}{+\left|{S}_{12}\right|}^{2}\right], {\xi }_{12}=-\left[{{S}_{11}}^{*}{{S}_{12}}+{{S}_{21}}^{*}{{S}_{12}}\right]$$

9 $${\xi }_{21}=-\left[{{S}_{22}}^{*}{{S}_{21}}^{ }+{{S}_{12}}^{*}{{S}_{21}}^{ }\right], {\xi }_{22}=1-\left[{\left|{S}_{22}\right|}^{2}{+\left|{S}_{21}\right|}^{2}\right]$$

### Radiation pattern

**Figure 12:**
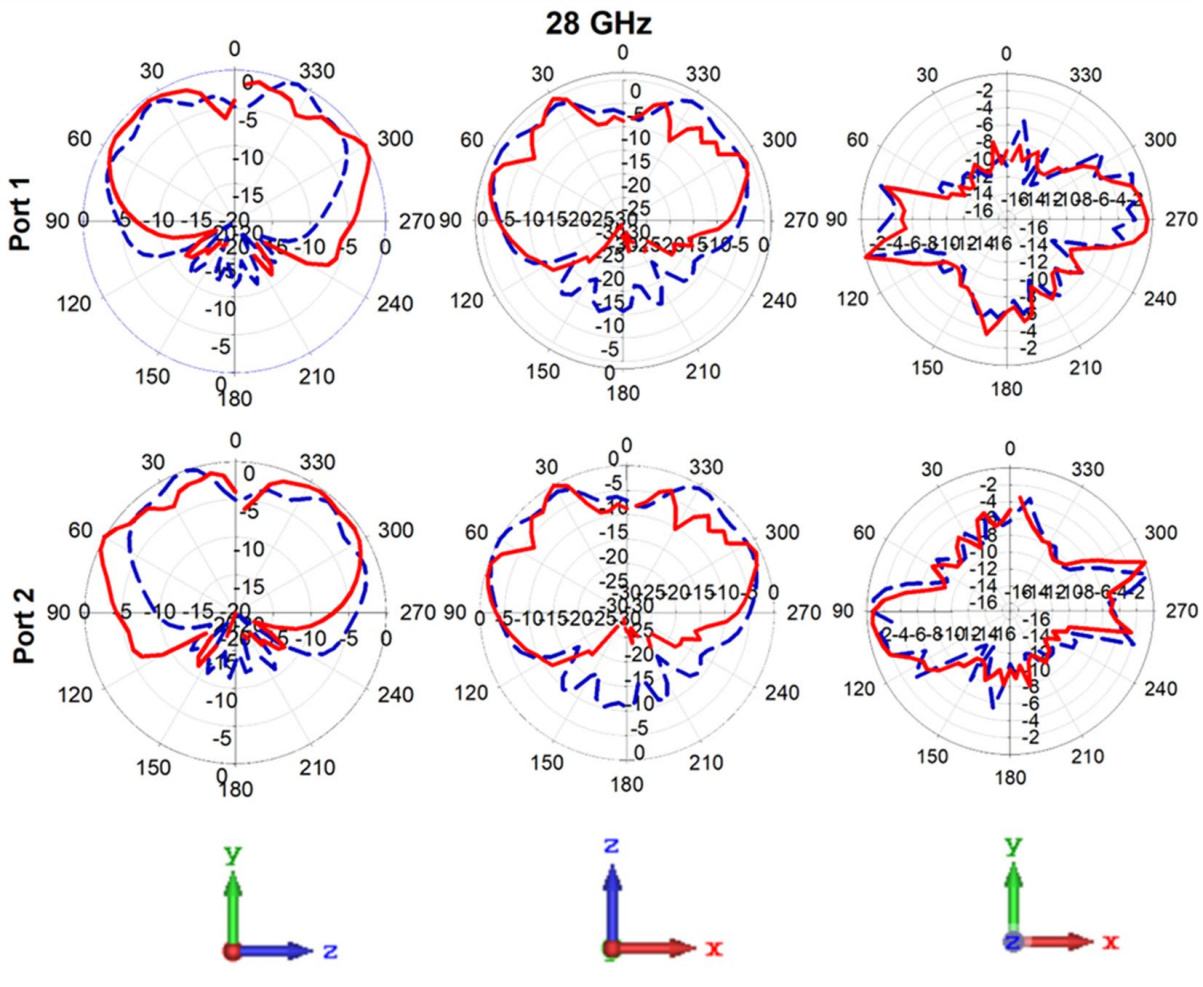
Radiation pattern of the proposed antenna system at 28 GHz.

Figure 12 exhibits the radiation patterns of the adjacent ports within the antenna system, as acquired through simulation and measurement. The Fig. 12 depicts MIMO antenna’s 2D radiation patterns for ports 1 and 2 were analyzed and compared in the E-plane along the YZ and XZ directions, as well as in the H-plane along the XY direction, with a specific orientation of phi (90°; 0°) in the YZ and XZ directions, and theta 90° in the XY direction. These patterns are observed at a frequency of 28 GHz. Based on the far-field observations of the antenna system, it is apparent that the primary beam is oriented perpendicular to the antenna, exhibiting discernible directional properties in both the YZ and XZ planes. However, slight disparities between the simulated and measured outcomes are noticeable in both planes as a result of limitations imposed by the measurement setup.

## Performance comparison

The efficacy of the proposed enhanced antenna system is evaluated by comparing it to recently published mm-wave MIMO antennas. Table 4 lists evaluation based on various performance metrics, including resonating bands, radiation efficiency, gain, bandwidth, isolation, ECC, DG, and the number of utilized ports and elements.

**Table 4:** Comparison to state-of-the-art of mm-wave MIMO antenna.

References	^7^	^26^	^27^	^28^	^29^	This study
Size (mm^2^)	30 × 35	20 × 20	75 × 110	3D	3D	30 × 200
No. of ports	4	2	6	12	32	16
No. of elements	8	2	10	12	32	64
BW (GHz)	4.1	1.34	3.5	3	1.5	3.5
Gain (dB)	8.3	8	9.53	6	8.17	19.9
Isolation (dB)	17	24	24	15	16	25
Efficiency (%)	80	–	73	95	90	99
ECC	0.01	0.013	–	–	–	0.008
Distance between elements (mm)	3.5	8.8	–	–	–	6.8

## Conclusion

This research proposes a 64-element based mMIMO antenna system loaded with metamaterial for future millimeter wave 5G applications. By utilizing the advantages of DNG/ENG metamaterials, the proposed antenna's isolation, gain, and efficiency have shown a significant enhancement with < 25 dB, 20 dBi, and 99% for isolation, maximum gain, and efficiency, respectively. DG and ECC characteristics have been examined and exhibited acceptable values. The suggested antenna was constructed and measured to confirm the design. The simulation's predictions match up well with the experimental findings. Unlike other reported studies, the proposed mMIMO antenna holds excellent potential for the upcoming 5G wireless communication systems.

## Data Availability

The datasets generated and/or analyzed during the current study are available from the corresponding author upon reasonable request.
