# Author Correction: High isolation 16-port massive MIMO antenna based negative index metamaterial for 5G mm-wave applications

**DOI:** 10.1038/s41598-024-59806-w

**Published:** 2024-04-25

**Authors:** Alya Ali Musaed, Samir Salem Al‑Bawri, Wazie M. Abdulkawi, Khaled Aljaloud, Zubaida Yusoff, Mohammad Tariqul Islam

**Affiliations:** 1 Space Science Centre, Climate Change Institute, Universiti Kebangsaan Malaysia (UKM), 43600 Bangi, Malaysia; 2 Department of Electrical Engineering, College of Engineering in Wadi Addawasir, Prince Sattam Bin Abdulaziz University, Al‑Kharj, Saudi Arabia; 3 College of Engineering, Muzahimiyah Branch, King Saud University, 11451 Riyadh, Saudi Arabia; 4 Faculty of Engineering, Multimedia University, 63100 Cyberjaya, Selangor Malaysia; 5 Department of Electrical, Electronic and Systems Engineering, Faculty of Engineering and Built Environment, Universiti Kebangsaan Malaysia UKM, 43600 Bangi, Selangor Malaysia

Correction to: *Scientific Reports* 10.1038/s41598-023-50544-z, published online 02 January 2024

In the original version of this Article, Samir Salem Al-Bawri was incorrectly affiliated with ‘Department of Electronics and Communication Engineering, Faculty of Engineering and Petroleum, Hadhramout University, 50512, Hadhramout, Al-Mukalla, Yemen.’

The correct Affiliation is listed below:

‘Space Science Centre, Climate Change Institute, Universiti Kebangsaan Malaysia (UKM), 43600, Bangi, Malaysia.’

The original Article has been corrected.

